# Life satisfaction of Taiwanese dental graduates received residencies in the U.S.: a cross-sectional study

**DOI:** 10.1186/s12909-020-02032-5

**Published:** 2020-04-28

**Authors:** Martin M. Fu, Rebecca Y. Chen, Huan-Chen Kao, Chi-Hsien Wang, Hsun-Liang Chan, Earl Fu, Tony Szu-Hsien Lee

**Affiliations:** 1 Department of Dentistry, Tri-Service General Hospital, National Defense Medical Center, Taipei, Taiwan; 2 Department of Dentistry, Taipei Tzu Chi Hospital, Buddhist Tzu Chi Medical Foundation, New Taipei City, Taiwan; 3 Private Practice, Taipei, Taiwan; 4 Department of Periodontics and Oral Medicine, School of Dentistry, University of Michigan, Ann Arbor, MI USA; 5 Department of Health Promotion and Health Education, College of Education, National Taiwan Normal University, Taipei, Taiwan

**Keywords:** Graduate dental education, Foreign professional personnel, Dental specialties, Dental residency, Satisfaction

## Abstract

**Background:**

Each year, more than 200 international dental graduates start U.S. specialty trainings to become specialists. It is unknown if their life satisfaction is associated with any dental career-related factor before residencies (e.g. dental school class rank, research experience, or private practice experience) and after residencies (e.g. staying in the U.S., teaching status, workplace, or board certification). This cross-sectional study aimed to identify these potential factors by surveying Taiwanese dental graduates who pursued U.S. residencies.

**Methods:**

Life satisfaction was measured with a structured questionnaire, Satisfaction With Life Scale (SWLS), which includes five statements on a 5-point Likert scale. Online surveys were sent out to 290 Taiwanese dental graduates who were known to pursue U.S. residencies. T-test, one way analysis of variance, and multivariable adjusted generalized linear model (GLM) were used to assess the differences of mean SWLS scores from different variables.

**Results:**

Surveys were completed by 158 dentists. Mean SWLS score of 125 specialists was higher (*p* = 0.0007) than the score of 33 residents. For the 125 specialists, multivariable adjusted GLM demonstrated better life satisfaction was positively associated with multiple independent factors, such as having research experience, being ranked in the top 26 ~ 50% of the class in dental school, starting U.S. residency within 4 years after dental school, starting residency before year 1996, and specializing in endodontics (vs. periodontics). Life satisfaction was not associated with any factors after residency (e.g. staying in the U.S. afterwards, teaching status, or workplace), but better mean life satisfaction score was significantly associated with being American specialty board certified (*p* < 0.001) for the specialists in the 26 ~ 75% of their class in dental school. For the 33 residents, better mean life satisfaction score was associated with better dental school class rank in both bivariate (*p* = 0.020) and multivariable adjusted GLM (*p* = 0.004) analyses.

**Conclusions:**

The life satisfaction of Taiwanese dental graduates pursuing U.S. residencies might be associated with some professional factors, such as research experience, dental school class rank, residency timing, specialty type, and specialty board certification. We hope our results may provide some objective information on making career decisions for international dental graduates/students who are preparing for U.S. residency.

## Background

Physicians’ well-being is critical not only to the physicians themselves, but also to their abilities to provide patient care [1]. Physicians with higher levels of well-being tend to provide better quality of patient care [2]. There is a vast amount of literature on the well-being of physicians [3–9], but the information on the well-being of dentists is limited [10].

Life satisfaction is a positive indicator of psychological well-being and is commonly defined as a cognitive assessment of satisfaction with one’s life circumstances [11]. Life satisfaction is commonly measured with a subjective self-reported questionnaire, the Satisfaction With Life Scale (SWLS) [12]. The SWLS is a rating designed to measure one’s satisfaction with their lives or happiness as a whole by one’s own standards, based on one’s own values and interests [12, 13].

According to the latest American Dental Association Survey of Advanced Dental Education year report [14], there are more than 200 international dental graduates starting various dental residency trainings in the U.S. every year. More than 40% of the prosthodontic residents and 30% of periodontal residents in the U.S. are also international dental graduates [14]. It is common to hear anecdotal evidence arguing the potential influences of some factors (such as specialty type, class rank in dental school, research experience, private practice experience, and the timing to start U.S. residency) on the career or life of an international dental graduate who received U.S. residency. However, it is unknown that if any of these above factors in the past could be related the life satisfaction of international dental graduates on completion or during his or her training program.

Certain specialties (e.g. orthodontics, pediatric dentistry, and endodontics) are known to be more competitive during the admission process for residency programs compared to other specialties (e.g. periodontics and prosthodontics) in the U.S. [14]. However, it is unknown if the specialists in those competitive specialties are more satisfied with their own lives than those in the less competitive specialties.

Dental school class rank has been well known to be one of the top three selection factors for the admission of U.S. dental residency programs [15–17]. Research experience and private practice experience are two other factors always evaluated during residency selection process [16–18]. However, the practicality of a good class rank, research experience and private practice experience of a dentist is also controversial, especially from points of view of general dentists and non-U.S. trained specialists.

After residency trainings, some residents chose to stay in the U.S. and/or take extra exams to become American specialty board certified. Whether staying in the U.S. or acquire American specialty board certification would result in a better life has also been discussed among dental specialists, but never been investigated scientifically.

To our best knowledge, there is only one previous study in the literature exploring the SWLS of dentists [19] which demonstrated that burnout and work engagement have effects on life satisfaction of Finnish dentists. This current study is the first study in the literature exploring the life satisfaction of dental specialists and residents.

This study aims to provide some insights on making career decisions for international dental graduates/students preparing U.S. residency trainings by assessing the dental career-related potential factors of life satisfaction among Taiwanese dental graduates who pursued specialty residencies in the U.S.

## Methods

### Measures

The SWLS questionnaire was initially developed in English [12]. In the present study, a 5-point Likert-scale ranging from 1 (strongly disagree) to 5 (strongly agree), which has been validated with Cronbach’s alpha coefficient of 0.82 [20], was used instead of original 7-point Likert-scale SWLS. The SWLS score of each sample was the sum of 5 items divided by 5 to have maximum of 5 and minimum of 1. Higher scores are indicative of greater satisfaction.

### Data collection

This cross-sectional study received the Institutional Review Board (IRB) approval from Tri-Service General Hospital, Taipei, Taiwan (No. 1–105–05-128). This study was exempt from IRB review from University of Michigan, Ann Arbor, USA (No. HUM00155739), because this study only involves survey procedures in such a manner that the identity of subjects cannot readily be ascertained, which would not place the subjects at risk.

The email account of any Taiwanese dental graduate who was known to pursue residency in the U.S. was obtained from U.S. and Taiwanese professional association member directories, dental school website faculty/resident directories, or through alumni. A total of 290 online surveys were sent out via Survey Monkey from January 2016 to May 2017.

The participants must have graduated from one of the seven dental schools in Taiwan and later attended Commission on Dental Accreditation (CODA)-accredited specialty programs in the U.S. to be included in the current study. CODA is recognized as the sole agency to accredit dental and dental-related education programs in the U.S. Therefore, those non-CODA accredited programs (e.g. fellowship or implant program) were excluded. Since the most common specialties in the U.S. for international dental graduates are endodontics, orthodontics, pediatric dentistry, periodontics, and prosthodontics, only the dentists who were in the above five specialties were included in the present study. Other specialties with very limited number of international dental graduates (such as oral surgery, oral pathology, oral medicine, orofacial pain, dental public health, etc.) were excluded.

In addition to SWLS, basic information (such as gender, specialty type of U.S. residency, date of enrollment) and dental career-related potential variables (such as class rank in dental school, any clinical training in Taiwan prior to U.S. residency, DDS/DMD degree prior to U.S. residency, prior research experience, and prior private practice experience) were also asked in the survey. For those whom identified themselves as specialists (i.e. those former residents who had finished their residency trainings), four more questions (including current living country, current teaching status, current major workplace, and American specialty board certification status) were also asked.

### Data analyses

Descriptive statistics (including frequency, mean and standard deviation) of collected variables were coded and analyzed using SPSS (IBM Inc., Chicago, IL, USA). Independent *t*-tests and one way analysis of variance (ANOVA) were used to assess the differences of mean SWLS scores from different variables. To examine the relative association of potential contributory variables on life satisfaction of specialists and residents, multivariable adjusted generalized linear model (GLM) was selected and used. In this multivariable model, each outcome of the examined dependent variables (including specialty type, residency started how long after dental school, the year residency started, class rank in dental school, prior research experience, prior practice experience, American board certification, and current country) is assumed to be generated from a particular distribution in an exponential family [21]. Significance level was set at *p* ≤ 0.05.

## Results

A total of 158 out of 290 (54.5%) dentists completed the online survey. Of the 158 dentists, 33 (20.9%) were still in residency trainings at the time of data collection. The average timing of U.S. residency enrollment was 4.01 years (median = 3; SD = 3.05; range 0–20) after graduating from dental school for those 125 specialists, and 4.21 years (median = 4; SD = 2.46; range 0–10) after graduating from dental school for those 33 current residents. The median year of starting U.S. residency was year 1999 (mean = 1997.5; SD = 10.5; range 1976–2012) for specialists, and year 2015 (mean = 2014.3; SD = 1.29; range 2012–2016) for current residents. Overall, the mean SWLS score was 4.01 (SD = 0.72), with significantly (*p* = 0.0007) higher mean score for specialists (4.11; SD = 0.69) than for current residents (3.64; SD = 0.70) (Table 1).

**Table 1:** Mean and Standard Deviation (SD) of SWLS by background of 158 participants

	Specialists / Former Residents						Current Residents					
	*N*	%	SWLS Mean SD		*F*	*p*-value	*N*	%	SWLS Mean SD		*F*	*p*-value
Gender					0.095	0.995					0.916	0.346
Male	76	60.8%	4.11	0.69			9	27.3%	3.44	0.50		
Female	49	39.2%	4.11	0.70			24	72.7%	3.71	0.76		
Dental School Class Rank					2.182	0.094					3.868	0.020*
Top 25%	92	73.6%	4.11	0.65			24	72.7%	3.75	0.61		
26 to 50%	16	12.8%	4.37	0.72			5	15.2%	3.72	0.78		
51 to 75%	4	3.2%	4.45	0.64			2	6.1%	2.30	0.42		
Bottom 25%	7	5.6%	3.68	0.40			1	3.0%	2.80			
Missing	6	4.8%					1	3.0%				
Specialty					2.293	0.063					0.305	0.872
Periodontics	54	43.2%	3.95	0.67			12	36.4%	3.73	0.82		
Prosthodontics	20	16.0%	4.20	0.68			11	33.3%	3.60	0.73		
Pediatric Dentistry	21	16.8%	4.05	0.75			3	9.1%	3.67	0.41		
Orthodontics	14	11.2%	4.21	0.65			3	9.1%	3.80	0.72		
Endodontics	16	12.8%	4.50	0.66			4	12.1%	3.30	0.62		
Clinical Training in Taiwan before Residency					0.066	0.797					2.238	0.145
No	89	71.2%	4.14	0.68			22	66.7%	3.77	0.76		
Yes	32	25.6%	4.11	0.61			11	33.3%	3.38	0.51		
Missing	4	3.2%										
U.S. DDS/DMD before Residency					0.789	0.376					0.269	0.608
No	111	88.8%	4.10	0.66			32	97.0%	3.63	0.71		
Yes	11	8.8%	4.29	0.89			1	3.0%	4.00			
Missing	3	2.4%										
Residency Started in					4.870	0.009**					0.027	0.974
0 ~ 4 years after dental school	84	67.2%	4.19	0.65		0.029*	20	60.6%	3.66	0.69		
5 ~ 9 years after dental school	33	26.4%	3.84	0.73			11	33.3%	3.60	0.81		
≥10 years after dental school	7	5.6%	4.54	0.51		0.032*	2	6.1%	3.60	0.57		
Missing	1	0.8%										
Year Residency Started					4.098	0.008**						
1976 to 1985	22	17.6%	4.36	0.77		0.013*						
1986 to 1995	36	28.8%	4.23	0.61								
1996 to 2005	28	22.4%	4.15	0.66		0.036*						
2006 to now	39	31.2%	3.81	0.67			33	100%	3.64	0.70		
Research Experience before U.S. Residency					3.023	0.085					0.320	0.576
No	81	64.8%	4.06	0.69			26	78.8%	3.60	0.70		
Yes	39	31.2%	4.29	0.58			7	21.2%	3.77	0.74		
Missing	5	4.0%										
Private Practice Experience before U.S. Residency					7.816	0.006**					0.053	0.819
No	28	22.4%	4.41	0.54			10	30.3%	3.68	0.64		
Yes	91	72.8%	4.02	0.67			23	69.7%	3.62	0.74		
Missing	6	4.8%										
Total	125		4.11	0.69			33		3.64	0.70		

SWLS Mean Score = (Item 1 + item 2 + Item 3 + item 4 + Item 5)/5

T-tests and one way analysis of variance with post hoc Tukey HSD test were performed on all participants to compare means of two groups and more than two groups respectively. Missing values were deleted from analysis. *p < 0.05; **p < 0 .01

For those 125 specialists (Table 1), there was no difference found on the mean SWLS score by gender, class rank in dental school, specialty, prior research experience, prior experience of advanced clinical training in Taiwan before U.S. residency, or having an U.S. DDS/DMD degree before U.S. residency. However, the specialists who started their U.S. residencies within 4 years and more than 10 years after graduating from dental school had significantly (*p* = 0.029 and 0.032, respectively) higher mean SWLS scores than those starting their U.S. residencies 5 to 9 years after dental school. The year of starting U.S. residency was also significantly (*p* = 0.008) associated with mean SWLS score. In addition, the specialists without any prior private practice experience before U.S. residency had significantly (*p* = 0.006) higher mean SWLS score than those with private practice experience. Current country (i.e. U.S. vs. Taiwan), current teaching status (i.e. full-time, part-time, or no teaching), major workplace (i.e. at private practice, hospital, or school), and whether or not American board certification was acquired, were not significantly associated with the mean SWLS score of those specialists (Table 2).

**Table 2:** Mean and SD of SWLS by current teaching status, practice setting, and board certification of 125 specialists

	*N*	%	SWLS Mean SD		*F*	*p*-value
Current Country					0.268	0.605
Practice in the U.S.	63	50.4%	4.13	0.72		
Practice in Taiwan	61	48.8%	4.07	0.67		
Elsewhere	1	0.8%				
Current Teaching Status					1.445	0.233
Full Time	34	27.2%	4.29	0.65		
Paid Part Time	20	16.0%	4.25	0.52		
Unpaid Part Time	21	16.8%	4.00	0.74		
No Teaching	44	35.2%	4.04	0.69		
Missing	6	4.8%				
Current Major Workplace					0.171	0.843
Private Practice	59	47.2%	4.11	0.69		
Hospital/School	46	36.8%	4.10	0.65		
Both Private & Hospital/School	16	12.8%	4.16	0.58		
Missing	4	3.2%				
American Board Certification					0.714	0.400
Board Certified	58	46.4%	4.19	0.66		
Board Eligible	62	49.6%	4.09	0.67		
Missing	5	4.0%				
Total	125		4.11	0.69		

One way ANOVA was performed on all participants to compare means of between groups. Missing values were deleted from analysis

**Fig. 1:**
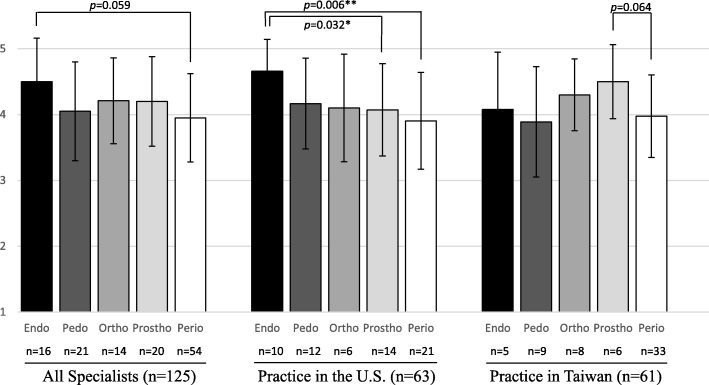
Means of SWLS by specialties and current location of 125 specialists. Endo = Endodontics; Pedo = Pediatric Dentistry; Ortho = Orthodontics; Prostho = Prosthodontics; Perio = Periodontics. ^*^p < 0.05; ^**^p < 0 .01

If further examining the 5 specialties separately according to which country they are practicing, the endodontists in the U.S. (Fig. 1; middle) had significantly (*p* = 0.032 and 0.006, respectively) higher mean SWLS score than the prosthodontists and periodontists in the U.S. However, for those 61 specialists who went back to Taiwan after their residencies (Fig. 1; right), there was no statistical difference on the mean SWLS scores among all five specialties. Within each specialty, the same specialty practice in the U.S. and Taiwan had no statistical difference on the mean SWLS scores (Fig. 1; middle vs. right).

Multivariable adjusted generalized linear model further showed that higher life satisfaction score from specialists was positively associated with the following factors: specialization in endodontics (vs. periodontics), enrollment of residency within 4 years after dental school graduation, enrollment of residency before the year 1996, ranking grade in the top 26 ~ 50% of the class in dental school, and prior research experience before U.S. residency (Table 3). Staying in the U.S. after completion of residency and receiving American board certification were again not associated with their mean life satisfaction score (*p* = 0.823). Higher SWLS score was no longer associated with absence of prior private practice experience (*p* = 0.397) or enrollment of residency more than 10 years after dental school graduation (*p* = 0.473) in the multivariable adjusted generalized linear model. However, endodontists had significantly (*p* = 0.001) higher mean SWLS score than the periodontists.

**Table 3:** Results of multivariable adjusted generalized linear model analysis of specialists: aspects of SWLS Mean Score

Intercept	*n*	%	B	95% CI		*P*-value
	116		3.410	2.886	3.934	0.000**
Specialty						0.026*
Prosthodontics	20	17.2%	0.173	−0.128	0.473	0.261
Orthodontics	13	11.2%	0.196	−0.176	0.569	0.301
Pediatric Dentistry	20	17.2%	−0.056	−0.362	0.251	0.721
Endodontics	13	11.2%	0.634	0.272	0.995	0.001**
Periodontics	50	43.1%	Referent	.	.	–
US Residency Started						0.004**
0 to 4 years after Graduation	82	70.7%	0.430	0.154	0.706	0.002**
≥10 years after Graduation	6	5.2%	0.203	−0.352	0.758	0.473
5 to 9 years after Graduation	28	24.1%	Referent	.	.	–
Year Residency Started						0.029*
1976 to 1985	20	17.2%	0.377	0.066	0.687	0.017*
1986 to 1995	33	28.4%	0.427	0.139	0.716	0.004*
1996 to 2005	27	23.3%	0.238	−0.061	0.537	0.119
2006 to now	36	31.0%	Referent	.	.	–
Class Rank in Dental School						0.058
Top 25%	89	76.7%	0.271	−0.170	0.711	0.219
26 to 50%	16	13.8%	0.556	0.051	1.060	0.031*
51 to 75%	4	3.4%	0.285	−0.436	1.007	0.438
Bottom 25%	7	6.0%	Referent	.	.	–
Research Experience						0.012*
No	80	69.2%	−0.295	−0.539	−0.051	0.012*
Yes	36	30.8%	Referent			–
Private Practice Experience						0.397
No	28	23.9%	0.099	−0.170	0.368	0.397
Yes	88	76.1%	Referent			–
American Board Certification						0.279
Board Eligible	60	52.1%	−0.127	−0.367	0.113	0.279
Board Certified	56	47.9%	Referent			–
Current Country						0.823
Practice in Taiwan	59	50.4%	0.025	−0.193	0.242	0.823
Practice in the U.S.	57	49.6%	Referent			–

*B* unstandardized beta, *SE B* standard error for the unstandardized beta; the adjusted variables listed in the table

^*^p < 0.05; ^**^p < 0 .01

**Fig. 2:**
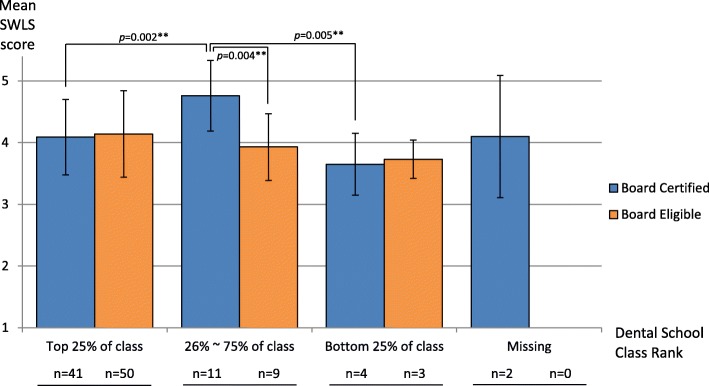
Relationship between dental school class rank and American board certification of 120 specialists. SWLS Mean Score = (Item 1 + item 2 + Item 3 + item 4 + Item 5)/5. One way ANOVA and independent T-test were performed on all participants to compare means of between groups. ^*^p < 0.05; ^**^p < 0 .01. Group of 26 to 50% and Group of 26 to 50% were combined due to lack of enough sample for analysis

Furthermore, the specialists who were not only board certified but also ranked in the 26 to 75% of their class in dental school (Fig. 2) had significantly higher mean SWLS score than all other groups. Analysis on the 33 current residents (Table 1; right column) showed no statistical difference on the mean SWLS score by any tested variables except their class rank in dental school (*p* = 0.020).

## Discussion

In the present study, we demonstrated that multiple factors (including prior research experience, being ranked in the top 26 ~ 50% of the class in dental school, enrollment in U.S. residency within 4 years after dental school graduation, and enrollment date of residency before the year 1996) may be independently associated with better current life satisfaction of US-trained dental specialists from Taiwan. However, whether they are living in the U.S. or Taiwan, holding a teaching position, or working at hospital/school or private practice may not be associated with life satisfaction.

To the best of our knowledge, the current study is the first study exploring the life satisfaction of dental specialists and residents. The mean SWLS score in the present study was consistent with the mean SWLS scores in the limited available literature on dentists [19], physicians, dental students, and medical students. Although the results from different Likert scales may not be compared, if we were able to convert the results from other related studies to the same common scale [22], the mean SWLS score of current residents in the current study would be similar to the mean SWLS scores of Finnish dentists [19], Brazilian physicians [23], medical/dental students in Saudi Arabia [24] [25], senior dental students in Saudi Arabia [26], medical students in New Zealand [27], China [28], India [29], and Malaysia [30]. However, the mean SWLS score of dental specialists in the current study was significantly higher than the mean SWLS scores of all of these above studies.

Using a single item questionnaire, two prospective cohort studies from the same group examined the life satisfaction of Norwegian first year medical students [31] and followed up on them for 15 years until 9 years after graduating from medical school [6]. The mean life satisfaction scores of medical students, medical residents, and early-career physicians in their studies were similar to the mean life satisfaction score of dental residents in the present study, but lower than the mean life satisfaction score of the dental specialists in the present study. Their study also showed that age, but not gender, is a factor of life satisfaction, which is consistent with our results in the present study.

We demonstrated that the specialists who received their residency trainings more than 20 years ago (e.g. in 1980s or 1990s) were more satisfied with their lives than those early-career specialists and residents. It is possible that the student loan or the associated financial stress from residency might be one of the potential causes, but further detailed study is needed to verify and investigate the causes. Our results are in accordance with the SWLS results from Brazilian physicians which showed that the oldest physicians had higher SWLS score than younger ones [32]. For retired U.S. orthopedic surgeons, a high level of life satisfaction has also been reported [7].

In the present study, we also found that the specialists who stayed in the U.S. and those who went back to Taiwan after their residencies had similar mean life satisfaction scores (Tables 2 and 3), and all five specialties showed no significant difference in mean SWLS scores between two different countries (Fig. 1). We hope our pilot results may provide some preliminary information for those considering staying in the U.S. to pursue more satisfied life or American dream.

Limited information could be found in the literature regarding class rank or GPA at tertiary education as an antecedent of life satisfaction. A cross-sectional study of Turkish dental students showed that SWLS score may be positively associated with their academic performance [33]. We demonstrated that the specialists who were ranked in the upper middle quartile (26 to 50%), but interestingly not top 25%, of their class in dental school had a significantly higher mean life satisfaction score than those in the bottom 25% of the class. Further study is definitely needed to confirm our results, but perhaps having a class rank of top 25% of the class may not be as beneficial as what most of people thought.

Board certification is an extra step that many specialists choose to take in order to demonstrate that they know the latest advancements in their specialty and to practice at the top of their profession. However, a previous study has showed that being certified by American Board of Orthodontics is non-significantly related to the job satisfaction of Canadian orthodontists [34]. We found that those specialists who were both American Board certified and also in the upper or lower middle quartile (26 to 75%) of the class had a significantly higher mean SWLS score than the specialists in any other group. Unfortunately, the sample size was too small to draw a conclusion, hence, further studies with larger sample size would be needed to explore and confirm these phenomenal findings.

We also demonstrated that the dental specialists who had research experience prior to their U.S. residencies had significantly (*p* < 0.012) higher life satisfaction than those without any prior research experience (Table 3). We hope our preliminary results could show dental students that having research experience could be associated with better future life satisfaction, and therefore encourage dental students and young dentists to participate in research projects. However, further detailed longitudinal research is still needed to verify such benefit of research experience on life satisfaction and explore the possible underlying reasons.

### Strengths and limitations

There are still limitations in the present study. Firstly, some cross-sectional studies have shown that physicians’ life satisfaction may be associated with many other factors which are not included in the current survey, such as health [5, 7, 8], personality/self-repair capacity [4, 6–8], being married/cohabitant [6, 8], a good sexual relationship [7, 8], social support [5, 6], stress [6, 8], recent major life events [5], job satisfaction [8], feelings of financial security [7], and have adequate resources for patient care [5]. More research may be requied to evalute whether or not these factors may influence a dentist’s life satisfaction when choosing to study abroad.

Secondly, socioeconomic or income factor is a factor not asked in the present study. However, it has been recently shown that the phenomenon that income is positively associate with life evaluation only occur at yearly income not more than USD$110,000 in East Asia and North America [35]. Dental specialists in Taiwan and the U.S. are known to have income higher than the above amount [36].

Thirdly, this study is limited to the international dental graduates who received dental degrees in Taiwan and specialty trainings in the U.S. without comparison groups. Therefore, the generalizability of the findings of this study to other dental populations remains unclear. Fourthly, due to the cross-sectional design, causality of the relationship between the exposure and outcome cannot be assumed.

There are less than 500 Taiwanese dentists who have attended U.S. residencies since 1970s. Only 290 online surveys were sent out due to lack of contact information of the rest of qualified candidates. Having 158 of them completed the survey as in this study, our descriptive data has covered a high percentage of the totally qualified candidates for the survey, and it is reasonable to assume that the results from this sample represent most of the U.S.-trained Taiwanese specialists. However, the absolute sample sizes from some of the variables (e.g. current resident or being ranked in the bottom 50% of the class in dental school) were too small to make subgroup comparisons.

Despite of all above limitations, we still sincerely hope that with the information from this study, the future Taiwanese dental graduates/students who have committed to U.S. residency could have more objective information about making decisions on their career or even life, and consequently their dental socialites and patients could benefit from their improved life satisfaction. However, future longitudinal studies with comparison groups are necessary to further verify our results and provide more insightful information.

## Conclusions

Multiple dental career-related independent factors before residency (such as having research experience before residency, being ranked in the top 26 ~ 50% of the class in dental school, starting U.S. residency within 4 years after dental school graduation, and starting residency before year 1996) may be positively associated with better life satisfaction of a U.S.-trained Taiwanese dental specialist. Those specialists who were both ranked in the 26 ~ 75% of their class in dental school and American specialty board certified had better life satisfaction than other groups. However, whether one chooses to stay in the U.S., hold a teaching position, and work at hospital/school or private practice, may not be related to the life satisfaction of Taiwanese dental graduates who have attended U.S. residencies.

## Data Availability

Additional data gathered in the survey and original datasets are available to any researcher who requests it from the authors.
